# Effect of non-stoichiometric Mn and Cr on the hydrogen storage properties of Ti–Mn-based alloys

**DOI:** 10.1039/d5ra00542f

**Published:** 2025-05-22

**Authors:** Qiuyang Pan, Hao Shen, Xingbo Han, Jianhui Zhu, Zhilin Li, Taijun Pan, Linhua Xu, Lijun Lv

**Affiliations:** a School of Mechanical and Energy Engineering, Zhejiang University of Science and Technology Hangzhou 310023 China tjpan2019@163.com; b Shanghai Institute of Applied Physics, Chinese Academy of Sciences Shanghai 201800 China xulinhua@sinap.ac.cn lvlijun@sinap.ac.cn; c School of Materials Science and Engineering, Changzhou University Changzhou 213164 China; d Shanghai CEO Environmental Protection Technology Co., Ltd. Shanghai 200082 China; e Shanghai Bolu Jie-an Amperex Technology Co., Ltd, . Shanghai 200082 China

## Abstract

The effects of non-stoichiometric Mn and Cr on the hydrogen storage properties of Ti–Mn-based Ti_0.84_Zr_0.16_Mn_0.9+*x*_Cr_0.7_Fe_0.1_ (*x* = 0, 0.1, 0.2) and Ti_0.84_Zr_0.16_Mn_0.9_Cr_0.7+*y*_Fe_0.1_ (*y* = 0, 0.1, 0.2, 0.3) alloys were investigated. The alloys were synthesized by arc melting and crystallized in a single C14-type Laves phase structure. With increasing Mn and Cr content, the equilibrium plateau pressures of the alloys during hydrogen absorption/desorption increased markedly, while the plateaus became flatter and the maximum hydrogen absorption capacities showed a slight decrease. Lower Mn/Cr ratios contribute to reduced hysteresis and lower plateau pressures. However, this improvement is associated with a decline in hydrogen storage capacity. The cyclic performance of the Ti_0.84_Zr_0.16_Mn_0.9_Cr_0.7_Fe_0.1_ alloy, which exhibited excellent hydrogen storage properties, was studied. It was found that the capacity retention rate reached 96.2% after 500 hydrogenation–dehydrogenation cycles.

## Abstract 2

In Ti–Zr–Mn–Cr–Fe alloys, increased non-stoichiometric Mn and Cr raises the equilibrium plateau pressure, flattens the plateau region, and slightly reduces hydrogen content. The optimized *x* = 0 with *y* = 0 alloy maintains 96.2% capacity retention after 500 hydrogenation–dehydrogenation cycles.

## Graphical abstract

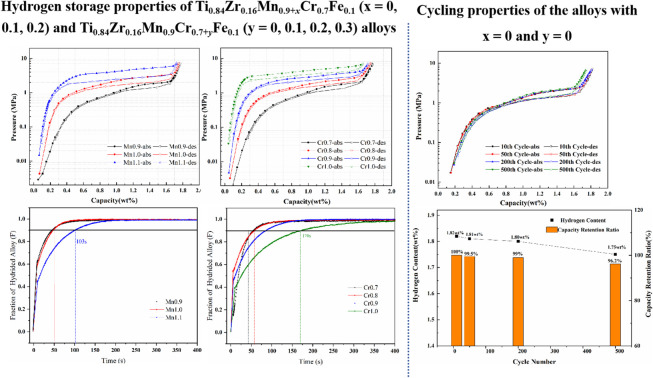

## 1. Introduction

With the continuous consumption of fossil fuel reserves and increasing environmental problems, efficient and clean sustainable energy has become essential. Hydrogen energy, due to its abundance and environmentally friendly nature, has attracted extensive attention from researchers.^1–5^ In hydrogen energy systems, hydrogen storage is especially important for the development of large-scale hydrogen applications. Currently, there are three main hydrogen storage technologies: high-pressure gaseous storage,^6^ low-temperature liquefaction storage,^7^ and solid-state storage.^8^ Compared with gaseous and liquefied methods, solid-state hydrogen storage offers several advantages, such as high energy density, good safety, lower cost, and good reversibility. Various hydrogen storage materials have been developed to better meet the requirements of solid-state hydrogen storage technology. These include hydrogen storage alloys (*e.g.*, rare-earth-based alloys^9^ and titanium-based alloys^10,11^), composite metal hydrides (*e.g.*, metal borohydrides^12,13^ and metal nitrides^14^), and light-metal hydrides (*e.g.*, magnesium hydrides^15^ and aluminum hydrides^16^). Among many hydrogen storage materials, Ti–Mn-based alloys have emerged as one of the most promising solid-state hydrogen storage materials due to their high reversible capacity, excellent hydrogen absorption/desorption kinetics, and relatively low cost.^17^ However, this series of alloys exhibits poor plateau hysteresis and limited cycle stability, which limit their application in solid-state hydrogen storage.^18^

Element substitution is one of the effective methods to improve the hydrogen storage performance of alloys. Due to the interstitial size effect, element substitution usually affects the lattice constants of alloy phases; the contraction or expansion of the lattice further alters the hydrogen absorption/desorption equilibrium pressures.^19^ Zr is often used to substitute Ti on the A-side. The atomic radius of Zr is larger than that of Ti; when increasing Zr content, the hydrogen absorption capacities increase and plateau pressures decrease.^20,21^ Zhou *et al.*^22^ studied the hydrogen storage properties of Ti_1−*x*_Zr_*x*_Mn_1.1_Cr_0.7_V_0.2_ (*x* = 0.05, 0.06, 0.07, 0.10) alloys, and the results showed that the partial substitution of Ti by Zr increases the hydrogen storage capacity and decreases the plateau pressure. In addition, Liu *et al.*^18^ found that the plateau slope of the alloy increases after Zr partially substitutes Ti, which can be explained by Ivey's theoretical model.^23^ In the C14-Laves structure, hydrogen atoms mainly occupy the A_2_B_2_ and AB_3_ interstitial spaces. Upon Zr partial substitution for Ti, the specific interstitial spaces become ZrZrB_2_, TiZrB_2_, TiTiB_2_, ZrB_3_ and TiB_3_. Due to the different affinity of Ti and Zr for hydrogen, the greater the Zr content, the greater the difference in amounts of interstice sites with different affinity, resulting in the more inclined plateau.^11^ On the other hand, a lot of studies on the substitution of Mn with Cr, Fe, Co, V, Cu, *etc.* on the B-side have been carried out. Bobet *et al.*^24^ studied the Ti_0.95_Zr_0.05_Mn_1.45_M_0.5_ (M = V, Cr, Mn, Co, Ni and Al) alloys and the results showed that Cr substitution slightly decreased hydrogen storage capacity and plateau pressure; in contrast, Ni substitution led to an increase in plateau pressure. Co substitution has almost no effect on hydrogenation kinetics, and Al substitution is conducive to reducing plateau pressure. However, it also resulted in a substantial decrease in hydrogen storage capacity. An investigation into the structures and hydrogen storage properties of Ti_0.9_Zr_0.1_Mn_1.8−*x*_Cr_*x*_V_0.2_ (*x* = 0.4, 0.8) alloys revealed that increasing the Cr content enlarges the cell volume and reduces hydrogen absorption/desorption hysteresis.^18^ Jiang *et al.*^25^ explored the effect of Fe doping on the hydrogen storage properties of TiCr_1.5−*x*_Mn_0.5_Fe_*x*_ (*x* = 0, 0.125, 0.25, 0.375, 0.5) alloys. It was found that the addition of Fe helps to increase the hydrogen storage capacity of the plateau, improve kinetic properties (the fastest hydrogen absorption at *x* = 0.375), but the hysteresis became worse. Bing *et al.*^26^ carried out a study on (Ti_0.8_Zr_0.2_)Mn_1.2_Cr_0.6−*x*_Ni_0.2_V_*x*_ (*x* = 0, 0.05) alloys, and the results showed that V could effectively reduce the plateau pressure while increasing the storage capacity. However, the cost of the alloy increased significantly due to more expensive V compared to Cr. By changing Mn/Cr ratio, Xu *et al.*^10^ prepared (Ti_0.85_Zr_0.15_)Mn_*y*_Cr_1.8−*y*_Fe_0.2_ (*y* = 1.00–0.40) alloys and found that the hydrogen storage capacity decreased with the decrease of Mn/Cr ratio, and the cell volume of the alloy decreased after Mn was substituted by Cr, resulting in the increase of hydrogen plateau pressure and hysteresis. Jiang *et al.*^27^ observed a similar phenomenon in the study of TiCr_2−*x*_Mn_*x*_ (*x* = 0, 0.25, 0.5, 0.75, 1) alloys.

Changing stoichiometric ratios can also improve hydrogen storage properties. Satoshi *et al.*^28^ prepared Ti-*x* at% Mn (*x* = 56, 57, 59, 60, 61, 62, 64, 67) alloys and found that the hydrogen storage capacity increases with increasing Mn content of the alloy up to 59.4%, and decreases rapidly with further increasing Mn content. Liang *et al.*^29^ also found a similar trend for Ti_1+*x*_Mn_2−*x*_ (*x* = 0.20, 0.25, 0.30, 0.35, 0.40 and 0.45) alloys. Huang *et al.*^30^ further proved that slight substoichiometry increased the maximum hydrogen storage capacity. In the study of TiCr_2*x*_(VFe)_*x*_ (*x* = 0.3–0.9), it was found that with the increase of (Cr + VFe)/Ti ratio, the plateau pressure gradually rises, the reversible hydrogen storage capacity increases first and then decreases, and (Cr + VFe)/Ti = 1.8 has the maximum reversible hydrogen storage capacity.

In this study, non-stoichiometric alloys Ti_0.84_Zr_0.16_Mn_0.9+*x*_Cr_0.7_Fe_0.1_ (*x* = 0, 0.1, 0.2) and Ti_0.84_Zr_0.16_Mn_0.9_Cr_0.7+*y*_Fe_0.1_ (*y* = 0, 0.1, 0.2, 0.3) were prepared. By varying the Mn and Cr content, the effects of Mn and Cr on the microstructure and hydrogen storage properties of the alloys were systematically investigated. This study provides new insights for optimizing the hydrogen storage properties of Ti–Mn-based hydrogen storage alloys.

## 2. Experimental

### 2.1. Alloy preparation

Ti_0.84_Zr_0.16_Mn_0.9+*x*_Cr_0.7_Fe_0.1_ (*x* = 0, 0.1, 0.2) and Ti_0.84_Zr_0.16_Mn_0.9_Cr_0.7+*y*_Fe_0.1_ (*y* = 0, 0.1, 0.2, 0.3) alloys were prepared by a vacuum arc furnace. The melting process was conducted in a water-cooled copper crucible under a 0.05 MPa Ar atmosphere (99.999%). The purity of the metals (Ti, Zr, Cr, Mn, Fe) was more than 99.9%. To ensure compositional uniformity, the alloy ingot was turned over and remelted three times. The oxide on the as-cast ingots' surface was removed by a small electric mill.

### 2.2. Microstructure and morphology analysis

The phase composition and crystal structure information of the alloys were collected by using X-ray diffraction (XRD) from Bruker with 2*θ* angle scanning range of 10° to 80°. The structural parameters and phase abundance were refined using TOPAS software through Rietveld refinement. The surface morphology of the alloy was observed by a Merlin compact scanning electron microscopy (SEM). The distribution of alloying elements was analyzed using EDS. In addition, a laser particle size analyzer (Malvern Mastersizer 2000) was used to determine the particle size of the alloy powder upon hydrogen absorption/desorption cycles.

### 2.3. Hydrogen absorption/desorption measurements

Pressure-composition temperature (PCT) curves were measured by an experimental apparatus based on the Sieverts method. Before PCT measurements, the 1.5 g samples were pretreated at room temperature under vacuum for 40 min, then activated upon three 7 MPa hydrogen absorption and desorption cycles. The PCT and kinetic measurements were conducted at 303 K, 318 K and 333 K respectively.

### 2.4. Cyclic properties

Hydrogen absorption/desorption cycles were performed at 298 K with a hydrogen pressure of 7 MPa and hydrogenation time of 15 min. To evaluate the cyclic stability of the alloys, PCT tests were performed at the 10th, 50th, 200th, and 500th cycles.

## 3. Results and discussion

### 3.1. Microstructure characterization

**Fig. 1:**
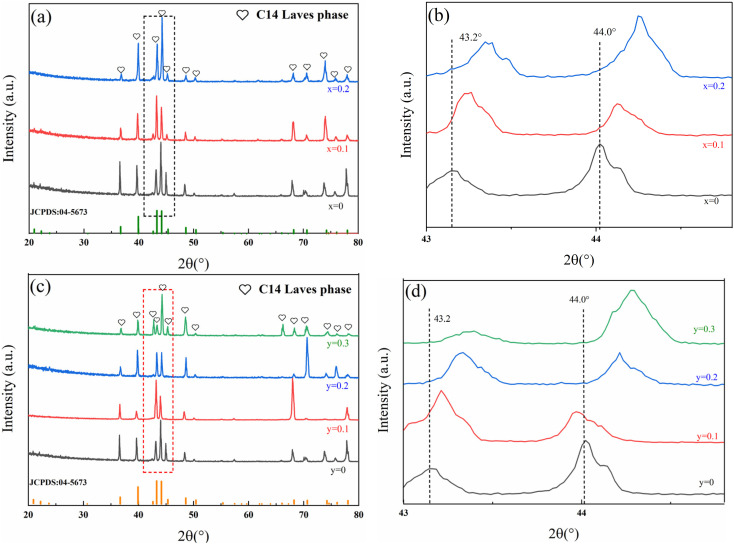
XRD patterns of the alloys: (a) and (b) Ti_0.84_Zr_0.16_Mn_0.9+*x*_Cr_0.7_Fe_0.1_ (*x* = 0, 0.1, 0.2) alloys and (c) and (d) Ti_0.84_Zr_0.16_Mn_0.9_Cr_0.7+*y*_Fe_0.1_ (*y* = 0, 0.1, 0.2, 0.3) alloys.

The XRD patterns of Ti_0.84_Zr_0.16_Mn_0.9+*x*_Cr_0.7_Fe_0.1_ (*x* = 0, 0.1, 0.2) and Ti_0.84_Zr_0.16_Mn_0.9_Cr_0.7+*y*_Fe_0.1_ (*y* = 0, 0.1, 0.2, 0.3) alloys are shown in Fig. 1(a) and (c). It was evident that all alloys maintained a single-phase C14-Laves structure. Fig. 1(b) shows a localized magnification between 43° and 45°. The XRD diffraction peaks shifted to the right as the Mn content increased. The cell parameters of the alloys obtained through Rietveld refinement are presented in Table 1. As the Mn content increased from 0.9 to 1.1, the lattice parameters, including *c*, as well as the cell volume, decreased to varying extents. Research on AB_2−*x*_La_0.03_ alloys (*x* = 0, 0.05, and 0.1; A = Ti_0.15_Zr_0.85;_ B = Mn_0.64–0.69_V_0.11–0.119_Fe_0.11–0.119_Ni_1.097–1.184_) has also reported the phenomenon of cell shrinkage with an increasing B/A stoichiometric ratio.^31^ It was found that the atomic radii of the elements in the A-site were usually larger than those of the elements in the B-site as the B/A stoichiometry increased. An excess of elements at the B-site occupied the A-site, leading to unit cell shrinkage.^31^ In this study, for the Ti_0.84_Zr_0.16_Mn_0.9+*x*_Cr_0.7_Fe_0.1_ alloy, the B/A ratio was 1.7 when *x* = 0, suggesting that A-site atoms (Ti, Zr) were displaced to B-site positions. As *x* increased, the B/A ratio increased correspondingly, causing the A-site atoms (Ti, Zr), which initially migrated to B sites, to return to their original A-site positions.^31^ Since the atomic radii were in the order of Zr (1.60 Å) > Ti (1.45 Å) > Mn (1.28 Å), the increase in Mn atoms replaced the Ti and Zr atoms that previously occupied the B-site positions, leading to a decrease in the cell volume.

**Table 1:** Lattice parameters of the Ti_0.84_Zr_0.16_Mn_0.9+*x*_Cr_0.7_Fe_0.1_ and Ti_0.84_Zr_0.16_Mn_0.9_Cr_0.7+*y*_Fe_0.1_ alloys

Sample	*a*/Å	*c*/Å	*V*/Å^3^
*x* = 0, *y* = 0	4.9007	8.0410	167.244
*x* = 0.1	4.8936	8.0337	166.613
*x* = 0.2	4.8845	8.0197	165.725
*y* = 0.1	4.8962	8.0361	166.848
*y* = 0.2	4.8904	8.0239	166.224
*y* = 0.3	4.8776	8.0118	165.135

From Fig. 1(d) and Table 1, it can be observed that the diffraction peak shifts to the right, and both the lattice parameter and cell volume decrease with increasing Cr content. As mentioned previously, the cell shrunk as the stoichiometric ratio on the B-site increased. This observation also applied to the study of non-stoichiometric Ti_0.84_Zr_0.16_Mn_0.9_Cr_0.7+*y*_Fe_0.1_ (*y* = 0, 0.1, 0.2) alloys. As the Cr content increased, the B/A ratio increased, and the A-site atoms (Ti, Zr) that previously occupied the B-site returned to their original A-site positions. Since the atomic radii were in the order of Zr (1.60 Å) > Ti (1.45 Å) > Cr (1.30 Å), the increase in Cr atoms replaced the Ti and Zr atoms that originally occupied the B-site, leading to a reduction in the cell volume.

By comparing the lattice parameters of Mn and Cr alloys with the same metrological ratio in Table 1, it was observed that the lattice constant and cell volume increased with a decrease in the Mn/Cr ratio. This was because Mn (1.28 Å) had a smaller atomic radius than Cr (1.30 Å), and an increase in Cr atoms occupied Mn's position, resulting in an increase in cell volume. Tu *et al.*^26^ also reached the same conclusion for (Ti_0.8_Zr_0.2_)_1.1_Mn_2−*x*_Cr_*x*_ (*x* = 0.8, 0.9, 1.0) alloys. As the Cr content increased, the Mn/Cr ratio decreased, and the diffraction peaks shifted to a lower angle, indicating that the lattice of the C14 phase expanded.

**Fig. 2:**
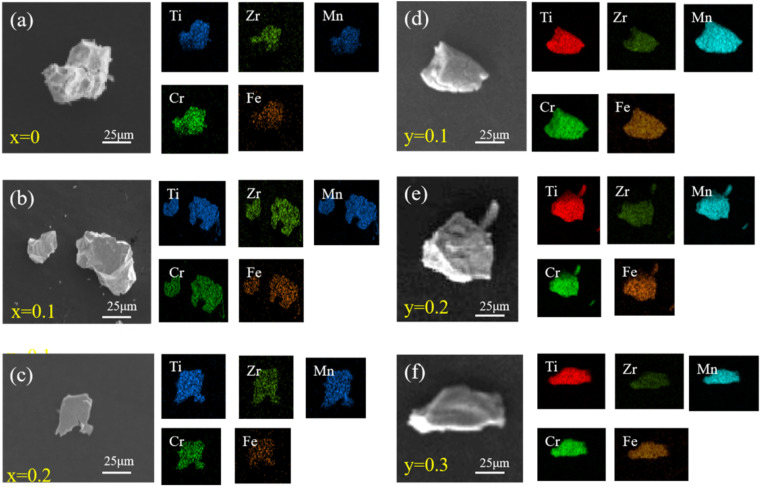
SEM micrographs and the EDS mapping of the Ti–Zr–Cr–Mn–Fe based alloys. (a)–(c) SEM images of the Ti_0.84_Zr_0.16_Mn_0.9+*x*_Cr_0.7_Fe_0.1_ (*x* = 0, 0.1, 0.2) alloys; (d)–(f) SEM images of the Ti_0.84_Zr_0.16_Mn_0.9_Cr_0.7+*y*_Fe_0.1_ (*y* = 0.1, 0.2, 0.3) alloys.

The surface morphology and elemental distribution of the Ti_0.84_Zr_0.16_Mn_0.9+*x*_Cr_0.7_Fe_0.1_ and Ti_0.84_Zr_0.16_Mn_0.9_Cr_0.7+*y*_Fe_0.1_ alloys were analyzed using SEM with EDS. As shown in Fig. 2, the EDS mappings indicated that Ti, Zr, Mn, Cr, and Fe were uniformly distributed.

### 3.2. Hydrogen storage properties

**Fig. 3:**
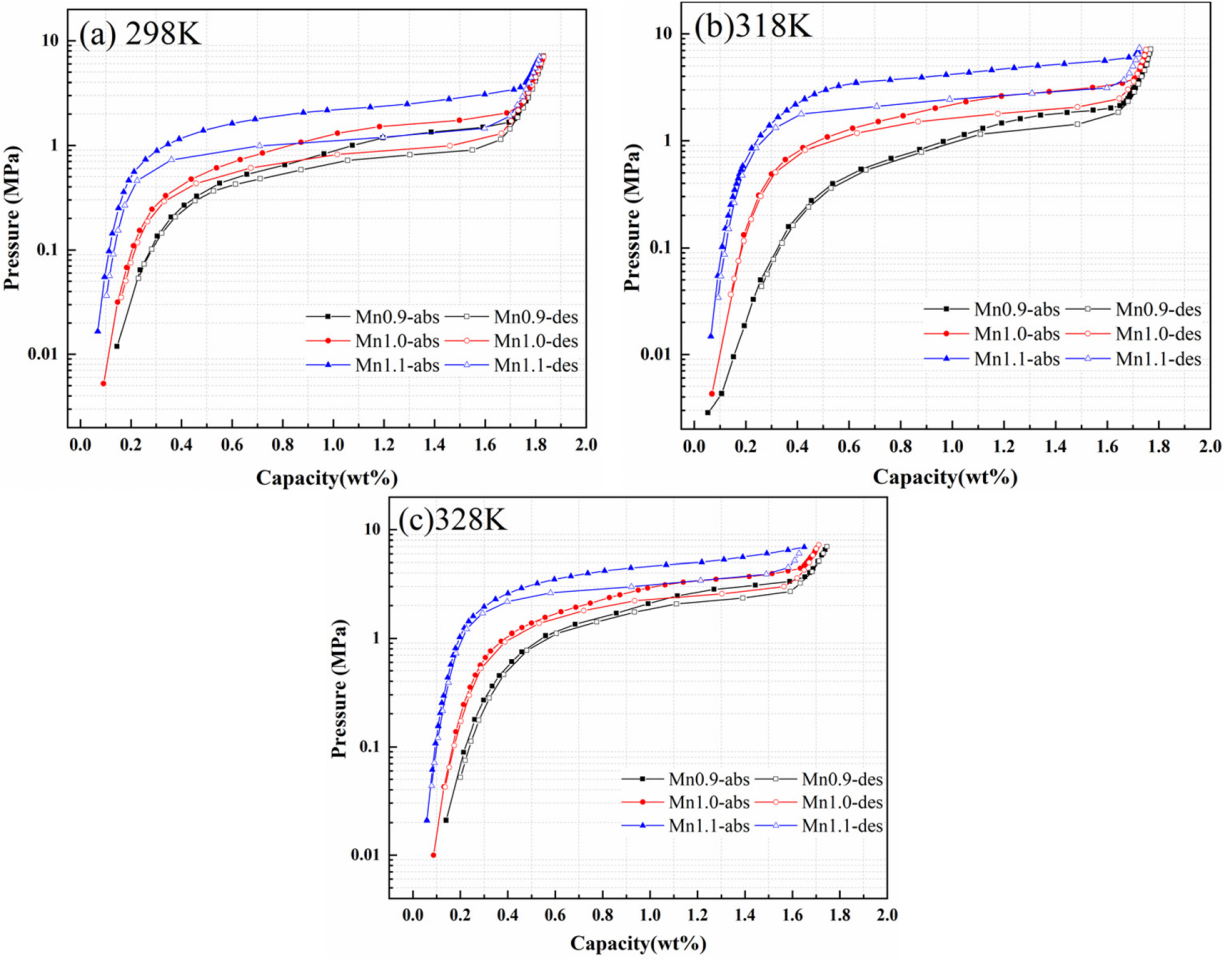
PCT curves of Ti_0.84_Zr_0.16_Mn_0.9+*x*_Cr_0.7_Fe_0.1_ (*x* = 0, 0.1, 0.2) alloys at (a) 298 K, (b) 318 K, and (c) 328 K.

The pressure-composition-temperature (PCT) curves and hydrogen storage performance parameters of Ti_0.84_Zr_0.16_Mn_0.9+*x*_Cr_0.7_Fe_0.1_ (*x* = 0, 0.1, 0.2) alloys at 298, 318,and 328 K under 7 MPa are shown in Fig. 3 and Table 2. With the increase in Mn content, the hydrogen storage capacity of the alloy decreased slightly, while the plateau pressures increased significantly. According to Liu *et al.*,^32^ for the non-stoichiometric Ti_0.84_Zr_0.16_Mn_0.9+*x*_Cr_0.7_Fe_0.1_ alloys, the phase was located on the Mn-rich side of the Laves phase due to the increase in Mn content. This resulted in a slight decrease in hydrogen storage capacity, as Ti had a greater affinity for hydrogen than Mn. The hydrogen storage capacity of the Ti_1+*x*_Mn_2−*x*_ (*x* = 0–0.45) alloy first increased and then decreased with the increase in Ti content. After partial substitution of Ti by Mn, the solid solution energy (*E*_sol_) at different interstitial positions increased, the cell volume decreased, and the plateau pressure increased. The alloy with a B/A ratio of 2 exhibited the highest hydrogen plateau pressure.^29^ According to the interstitial size effect described by Lundin *et al.*,^19^ the radius of Mn atoms on the B-site was smaller than that of Ti and Zr on the A-site. As a result, an increase in Mn content reduced the cell volume. This reduction in cell volume decreased the storage space available for hydrogen atoms, making it more challenging for hydrogen to penetrate the crystal structure. In the present study, the reduction in cell volume caused by Mn substitution resulted in an increase in plateau pressure. Peng *et al.*^33^ investigated Ti_1.04+*x*_Cr_2−*y*−*z*_Mn_*y*_Fe_*z*_ (*x* = 0.02, 0.04, 0.06, *y* = 0.2, 0.3, 0.4, *z* = 0.5, 0.6, 0.7) alloys and similarly observed that increasing Mn content elevated the plateau pressure—a trend attributed to interstitial size effects.

**Table 2:** Hydrogen storage characteristics of the Ti_0.84_Zr_0.16_Mn_0.9+*x*_Cr_0.7_Fe_0.1_ and Ti_0.84_Zr_0.16_Mn_0.9_Cr_0.7+*y*_Fe_0.1_ alloys

Alloy	*T*/K	*P*_abs_/MPa	*P*_des_/MPa	*H*_f_	Slope	*C*_max_/wt%
*x* = 0	**298**	0.92	0.64	0.36	3.02	1.83
*y* = 0	303	1.21	0.86	0.25	3.14	1.82
	318	1.91	1.52	0.23	3.23	1.77
	328	2.43	2.26	0.19	3.34	1.75
	333	2.81	2.40	0.16	3.49	1.71
*x* = 0.1	**298**	1.77	1.03	0.54	1.82	1.83
	318	3.03	2.05	0.39	1.94	1.75
	328	3.85	2.84	0.30	1.98	1.71
*x* = 0.2	**298**	2.86	1.39	0.72	1.07	1.81
	318	5.37	2.94	0.60	1.14	1.72
	328	6.17	3.96	0.44	1.18	1.65
*y* = 0.1	**303**	1.67	1.27	0.27	1.87	1.80
	318	2.65	2.05	0.26	2.05	1.75
	333	3.85	3.31	0.15	2.32	1.68
*y* = 0.2	**303**	2.57	1.77	0.37	1.31	1.79
	318	3.86	2.86	0.30	1.34	1.71
	333	5.67	4.81	0.16	1.54	1.63
*y* = 0.3	**303**	3.86	2.53	0.42	0.64	1.76
	318	5.86	4.28	0.31	0.66	1.65
	333	—	—	—	0.97	1.27

**Fig. 4:**
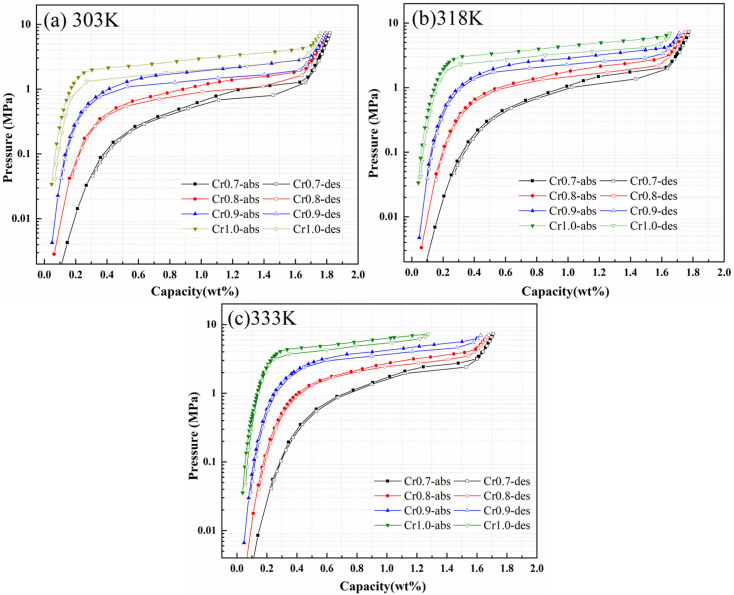
PCT curves of Ti_0.84_Zr_0.16_Mn_0.9_Cr_0.7+*y*_Fe_0.1_ (*y* = 0, 0.1, 0.2, 0.3) alloys measured at (a) 303 K, (b) 318 K, and (c) 333 K.

Fig. 4 and Table 2 present the PCT curves and hydrogen storage characteristics for Ti_0.84_Zr_0.16_Mn_0.9_Cr_0.7+*y*_Fe_0.1_ (*y* = 0, 0.1, 0.2, 0.3) alloys at 303 K, 318 K, and 333 K. It could be observed that the hydrogen storage capacity decreased with increasing Cr content, while the plateau pressure increased significantly. Li *et al.*^34^ also observed that the alloy plateau pressure decreased as the B-site stoichiometric ratio decreased. Alexander *et al.*,^35^ in their study of TiCr_2−*x*_ (*x* = 0.15–0.45) alloys, found that hydrogen content decreased and the plateau pressure increased with increasing Cr content. According to the microstructure analysis mentioned earlier, the decrease in hydrogen storage capacity with increasing Cr content was related to the alloy's phase composition being located on the Cr-rich side of the Laves phase. According to Lundin's interstitial size effect,^19^ the increase in Cr content decreased the cell volume, and the number of sites that could accommodate hydrogen atoms decreased, resulting in an elevated plateau pressure for Ti_0.84_Zr_0.16_Mn_0.9_Cr_0.7+*y*_Fe_0.1_ alloys.

**Fig. 5:**
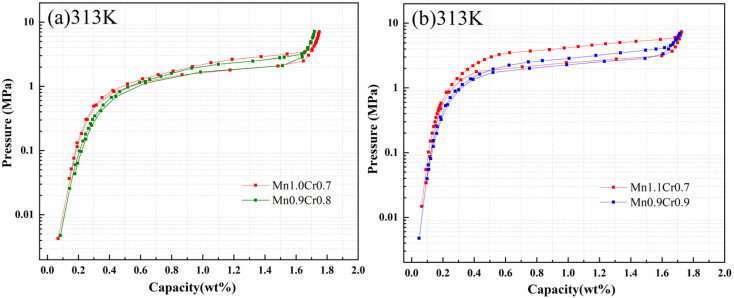
PCT curves of Ti_0.84_Zr_0.16_Mn_0.9+*x*_Cr_0.7+*y*_Fe_0.1_ at 313 K: (a) *x* = 0.1, *y* = 0 and *x* = 0, *y* = 0.1 (b) *x* = 0.2, *y* = 0 and *x* = 0, *y* = 0.2.

Fig. 5 and Table 2 display the PCT curves and hydrogen storage properties of two alloy series, each maintaining identical stoichiometric ratios of Mn and Cr. With the replacement of Mn by Cr, the plateau pressure of the alloys decreased, and the hydrogen content decreased slightly. Zhou *et al.*^22^ found that for Ti_0.95_Zr_0.05_Mn_1.8−*y*_Cr_*y*_V_0.2_ (*y* = 0.5–0.9) alloys, both the plateau pressure and hydrogen storage capacity decreased slightly with decreasing Mn/Cr ratio. This was because Mn promoted the formation of the hydride phase and reduced the plateau pressure,^27,36^ but the hydrogen storage capacity decreased with increasing Cr substitution. As seen in Table 1, Cr substitution for Mn increased the cell volume of the alloy, leading to a decrease in the plateau pressure. Table 3 lists the hydrogen storage properties of Ti–Mn-based alloys reported by other researchers. It can be observed that, as a material for hydrogen storage applications, the alloy in this work exhibits a more suitable plateau pressure and higher hydrogen storage capacity.

**Table 3:** Hydrogen storage characteristics of several other alloys reported in the literature

Alloy	Temperature (K)	*P*_des_ (MPa)	Capacity (wt%)	References
Ti_0.84_Zr_0.16_Mn_0.9_Cr_0.7_Fe_0.1_	298	0.64	1.83	This work
Ti_0.92_Zr_0.10_Cr_1.0_Mn_0.6_Fe_0.4_	363	24.91	1.74	37
(Ti_0.85_Zr_0.15_)_1.02_Mn_0.4_Cr_1.4_Fe_0.2_	283	1.04	1.80	10
(Zr_0.7_Ti_0.3_)_1.04_Fe_1.8_V_0.2_	273	1.12	1.51	38
(Ti_0.85_Zr_0.15_)_1.1_Cr_0.925_MnFe_0.075_	273	1.06	1.54	39
(Ti_0.85_Zr_0.15_)_1.1_Cr_0.9_Mo_0.1_Mn	273	0.95	1.78	40

According to the formula *H*_f_ = ln(*P*_a_/*P*_d_), the hysteresis coefficients of the Ti_0.84_Zr_0.16_Mn_0.9+*x*_Cr_0.7_Fe_0.1_ and Ti_0.84_Zr_0.16_Mn_0.9_Cr_0.7+*y*_Fe_0.1_ alloys at different temperatures were calculated, where *P*_a_ and *P*_d_ denote the hydrogen absorption and desorption pressures, respectively. The calculated results are shown in Table 2, where the hysteresis coefficients *H*_f_ of the alloys increased with *x* = 0–0.2 and *y* = 0–0.3. The hysteresis was primarily caused by the stresses that occurred during the growth of metal hydride nuclei.^41^ In this study, the gradual decrease in cell size with the increase in Mn and Cr content led to an increase in the volume change and internal stresses during the hydrogenation and dehydrogenation processes, thereby increasing the hysteresis of the alloys. From Table 2, it can be observed that the hysteresis coefficient gradually decreases as the temperature rises from 303 K to 333 K. This is due to the expansion of the crystal lattice upon temperature increase, which provides more space to accommodate hydrogen and reduces the lattice stress caused by lattice expansion.^42^ The plateau slope of the alloys decreased with the increase in Mn and Cr content, which was consistent with the pattern of change in the plateau width observed in the PCT curves.

### 3.3. Thermodynamic properties

**Fig. 6:**
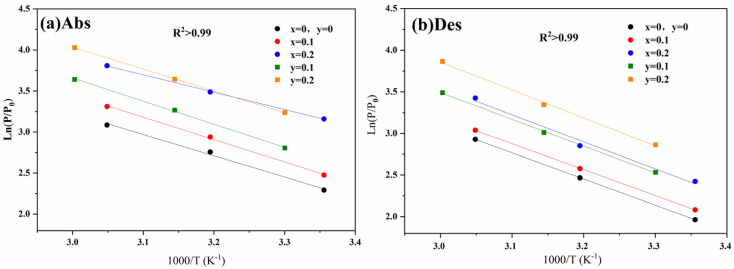
Van't Hoff curves for Ti_0.84_Zr_0.16_Mn_0.9+*x*_Cr_0.7_Fe_0.1_ and Ti_0.84_Zr_0.16_Mn_0.9_Cr_0.7+*y*_Fe_0.1_ alloys: (a) absorption and (b) desorption.

Fig. 6 shows the Van't Hoff curves of Ti_0.84_Zr_0.16_Mn_0.9+*x*_Cr_0.7_Fe_0.1_ and Ti_0.84_Zr_0.16_Mn_0.9_Cr_0.7+*y*_Fe_0.1_ alloys, and the thermodynamic parameters of the alloys were calculated and shown in Table 4. The value of Δ*H*_abs_ for the Mn series alloys ranged from −18.8 kJ mol^−1^ to −21.5 kJ mol^−1^, and for the Cr series alloys, it ranged from −20.8 kJ mol^−1^ to −21.5 kJ mol^−1^. The absolute values of enthalpy changes (Δ*H*_abs_ and Δ*H*_des_) of the alloys decreased with the increase in Mn and Cr content, indicating that the stability of the metal hydrides was decreasing. From Table 4, it could be seen that the replacement of Cr by Mn decreased the Δ*H* of the alloy, making the hydride system less stable. By comparing Tables 1 and 4, it was found that the Δ*H*_abs_ and Δ*H*_des_ of the alloys increased with the increase in cell volume, illustrating the correlation between the cell size of the alloys and the enthalpy of hydride formation. Li *et al.*^43^ proposed that the enthalpy of hydride formation depends mainly on two factors: the electronegativity factor and the electron density parameter. The electronegativity factor is related to the volume contraction from the pure metal to the alloy, while the electron density parameter is related to the molar volume. Due to these dependencies, the enthalpy is proportional to the cell volume. In the present work, the cell volume of the alloy decreased with increasing Mn and Cr content, and the enthalpy of hydride formation decreased. This conclusion was supported by the studies of Cao and Park *et al.*^40,44^

**Table 4:** Thermodynamic parameters of the Ti_0.84_Zr_0.16_Mn_0.9+*x*_Cr_0.7_Fe_0.1_ and Ti_0.84_Zr_0.16_Mn_0.9_Cr_0.7+*y*_Fe_0.1_ alloys

Alloy	Δ*H*_abs_/kJ mol^−1^	Δ*H*_des_/kJ mol^−1^	Δ*S*_abs_/J mol^−1^ K^−1^	Δ*S*_des_/J mol^−1^ K^−1^
*x* = 0, *y* = 0	−21.5	26.1	−89.2	99.1
*x* = 0.1	−20.4	25.9	−88.8	106.8
*x* = 0.2	−18.8	25.2	−88.3	107.6
*y* = 0.1	−21.2	26.0	−90.9	105.2
*y* = 0.2	−20.8	25.9	−95.7	106.9

### 3.4. Kinetic properties

**Fig. 7:**
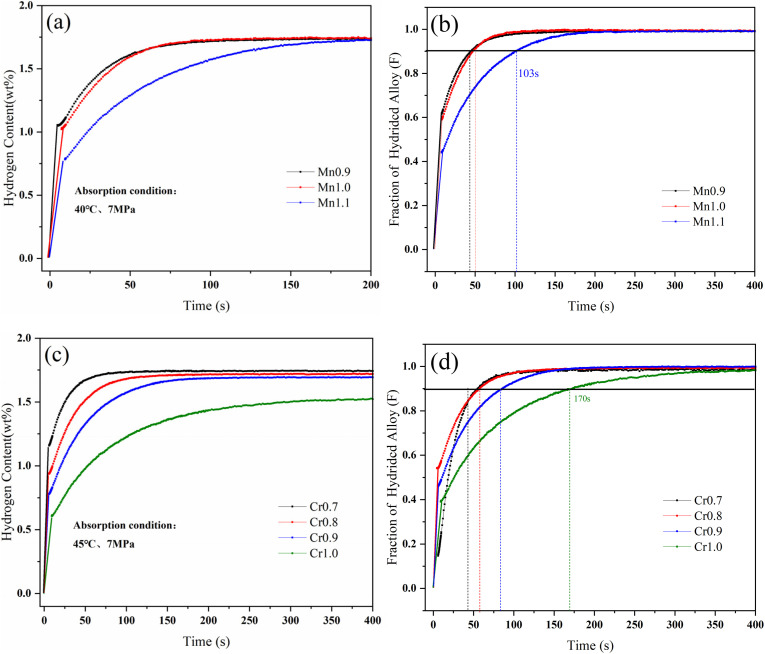
Hydrogen absorption kinetic curves of the alloys. (a) Ti_0.84_Zr_0.16_Mn_0.9+*x*_Cr_0.7_Fe_0.1_ alloys at 313 K, (b) normalized curves, (c) Ti_0.84_Zr_0.16_Mn_0.9_Cr_0.7+*y*_Fe_0.1_ alloys at 318 K, (d) normalized curves.

The hydrogen absorption kinetic curves of Ti_0.84_Zr_0.16_Mn_0.9+*x*_Cr_0.7_Fe_0.1_ (*x* = 0, 0.1, 0.2) and Ti_0.84_Zr_0.16_Mn_0.9_Cr_0.7+*y*_Fe_0.1_ (*y* = 0, 0.1, 0.2, 0.3) alloys are shown in Fig. 7. All alloys were able to reach 90% of their hydrogen storage capacity within 180 s, demonstrating excellent hydrogen absorption kinetics. From Fig. 7(b) and (d), it can be observed that the hydrogen absorption rate decreases with increasing Mn and Cr content. In the case of Mn substitution, the kinetics of the alloys with *x* = 0 and *x* = 0.1 did not change significantly. The Chou model was used to analyze the kinetics of the alloys.^45^ The expression of the Chou model is as follows:1
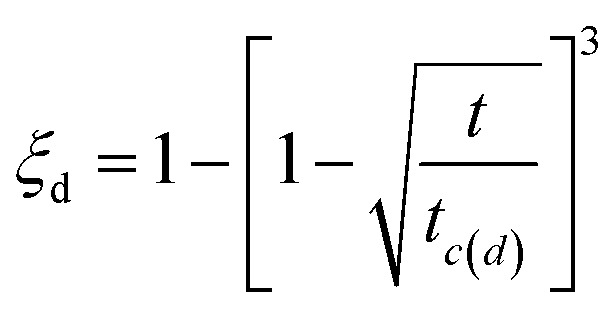


**Fig. 8:**
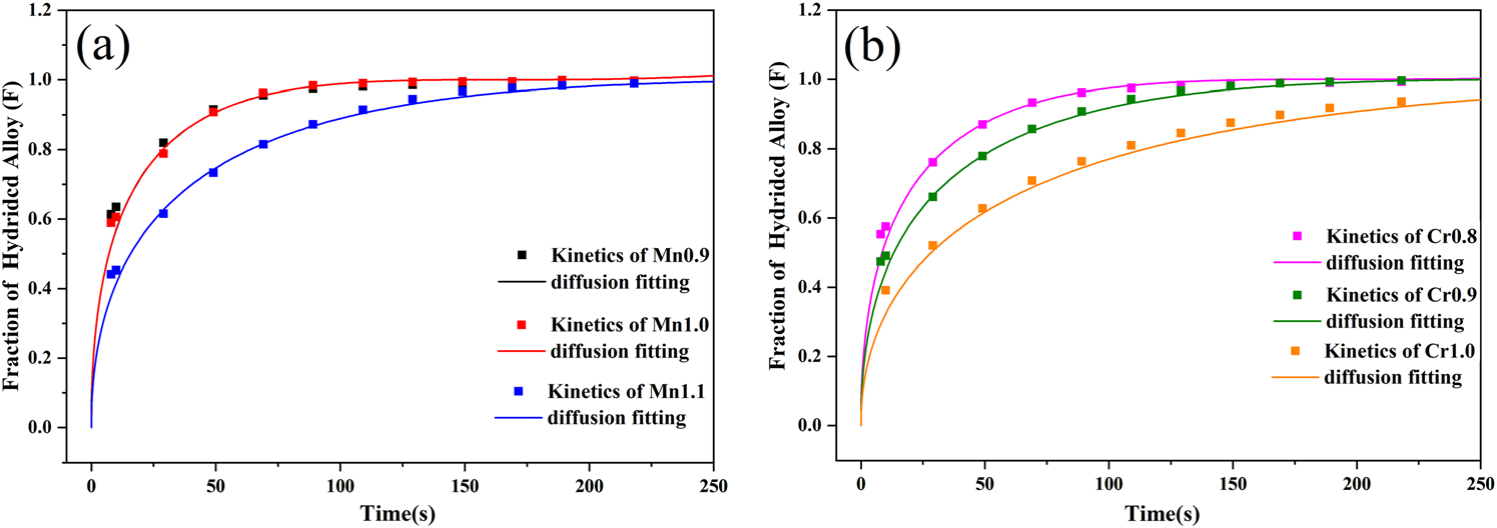
The fitted hydrogen absorption kinetics curves of the alloys: (a) Ti_0.84_Zr_0.16_Mn_0.9+*x*_Cr_0.7_Fe_0.1_, (b) Ti_0.84_Zr_0.16_Mn_0.9_Cr_0.7+*y*_Fe_0.1_.

The kinetic fitting diagrams of the alloys Ti_0.84_Zr_0.16_Mn_0.9+*x*_Cr_0.7_Fe_0.1_ and Ti_0.84_Zr_0.16_Mn_0.9_Cr_0.7+*y*_Fe_0.1_ are shown in Fig. 8. The fitting and experimental results were in good agreement, indicating that the controlling step of the hydrogen absorption kinetics for both Ti_0.84_Zr_0.16_Mn_0.9+*x*_Cr_0.7_Fe_0.1_ and Ti_0.84_Zr_0.16_Mn_0.9_Cr_0.7+*y*_Fe_0.1_ alloys was the diffusion control of hydrogen atoms in the metal hydrides. The kinetic simulation by Jiang *et al.*^27^ showed that the hydrogen absorption rate of the alloy was inversely proportional to the plateau pressure and particle size and directly proportional to the diffusion coefficient of hydrogen in the metal. Zhang *et al.*^46^ studied Zr_0.9_Ti_0.1_V_*x*_ (*x* = 1.7–2.3) alloys and found that the low-stoichiometric Zr_0.9_Ti_0.1_V_1.7_ alloy had the lowest plateau pressure and faster hydrogenation kinetics. As demonstrated in the previous section, the plateau pressure increased with the addition of Mn and Cr. This indicated that the hydrogen uptake rate of the alloy declined as the plateau pressure increased.

### 3.5. Hydrogen cycling properties

**Fig. 9:**
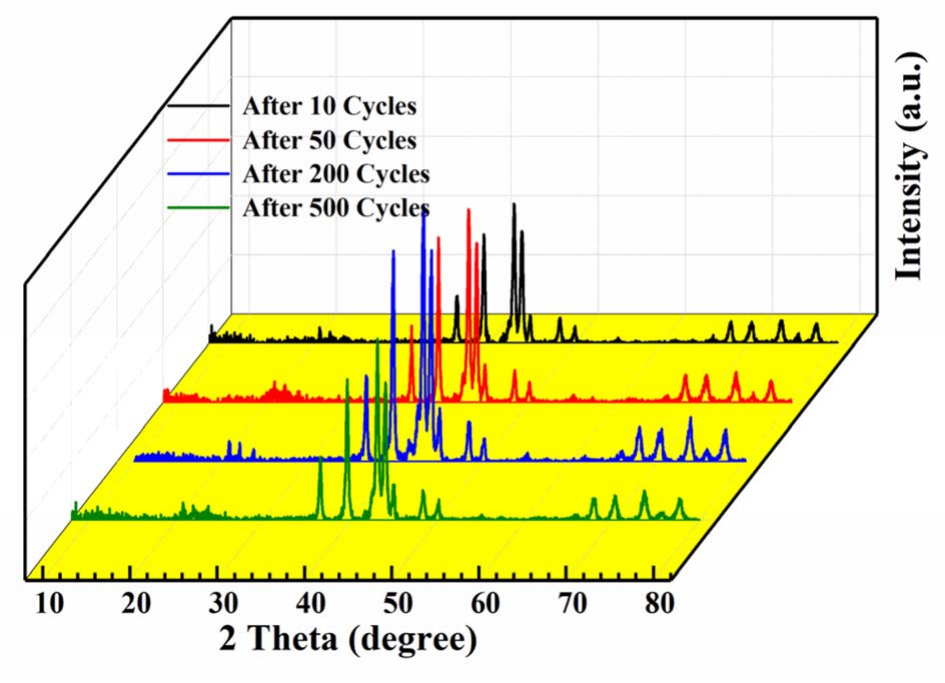
XRD patterns of the Ti_0.84_Zr_0.16_Mn_0.9_Cr_0.7_Fe_0.1_ alloy after different cycle numbers.

**Fig. 10:**
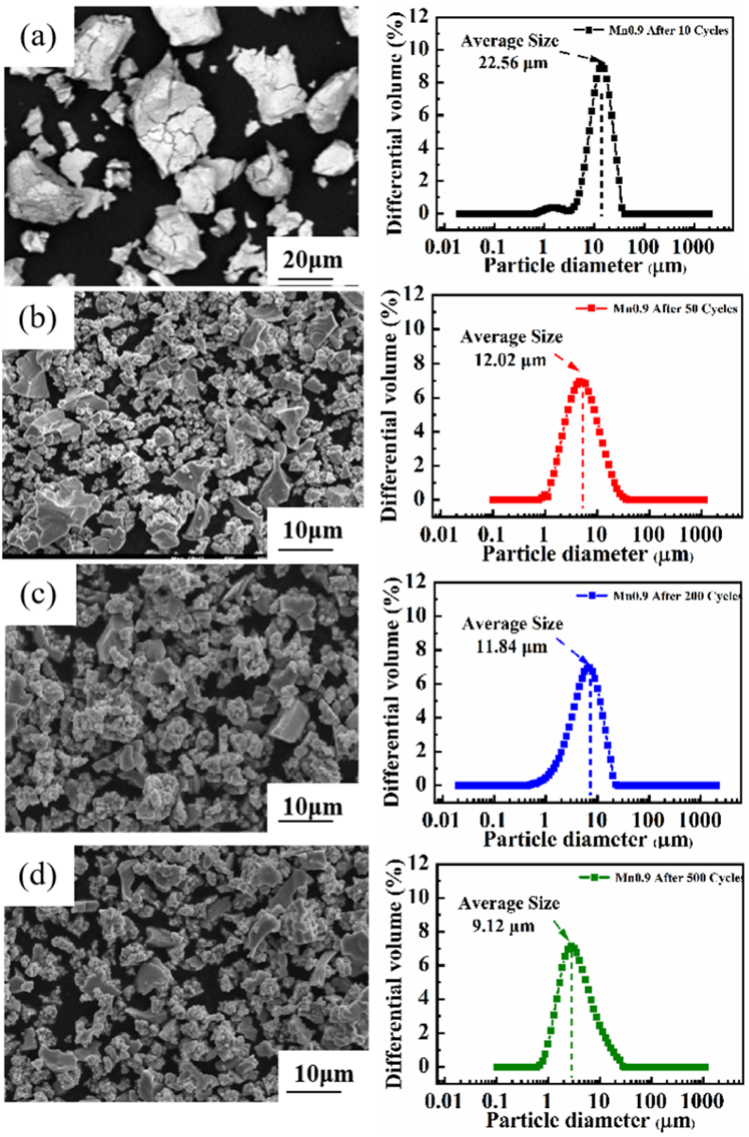
SEM images and size distribution plots of the Ti_0.84_Zr_0.16_Mn_0.9_Cr_0.7_Fe_0.1_ alloy at different cycle numbers.

Among the Ti_0.84_Zr_0.16_Mn_0.9+*x*_Cr_0.7_Fe_0.1_ (*x* = 0, 0.1, 0.2) alloys, the Ti_0.84_Zr_0.16_Mn_0.9_Cr_0.7_Fe_0.1_ alloy exhibited superior hydrogen storage properties. Therefore, this alloy was selected for further exploration of its cyclic stability. To study the phase structure evolution of the alloy, it was analyzed by XRD after 500 cycles. Fig. 9 shows the XRD patterns of the Ti_0.84_Zr_0.16_Mn_0.9_Cr_0.7_Fe_0.1_ alloy after 10, 50, 200, and 500 cycles. The alloy maintained its original phase composition after cycling, but the peak intensity decreased and the peak width increased from the 200th to the 500th cycle, which was likely due to increased micro-strain, grain refinement, or even non-crystallization.^47^ To examine the changes in alloy particle size during cycling, the particle size distribution curves of the Ti_0.84_Zr_0.16_Mn_0.9_Cr_0.7_Fe_0.1_ alloy after various cycles are presented in Fig. 10. The particle size decreased with an increase in the number of cycles. The alloy particle size decreased from 22.56 μm after 10 cycles to 9.12 μm after 500 cycles. This was due to the lattice stress generated by the hydrogen atoms moving in and out of the crystal structure. Lattice stress can cause microcracking and lead to the pulverization of alloy particles. It can also be observed from Fig. 10 that the comminution of the alloy occurred mainly in the first 50 cycles, and the particle size reduction was not significant in the subsequent cycles.

**Fig. 11:**
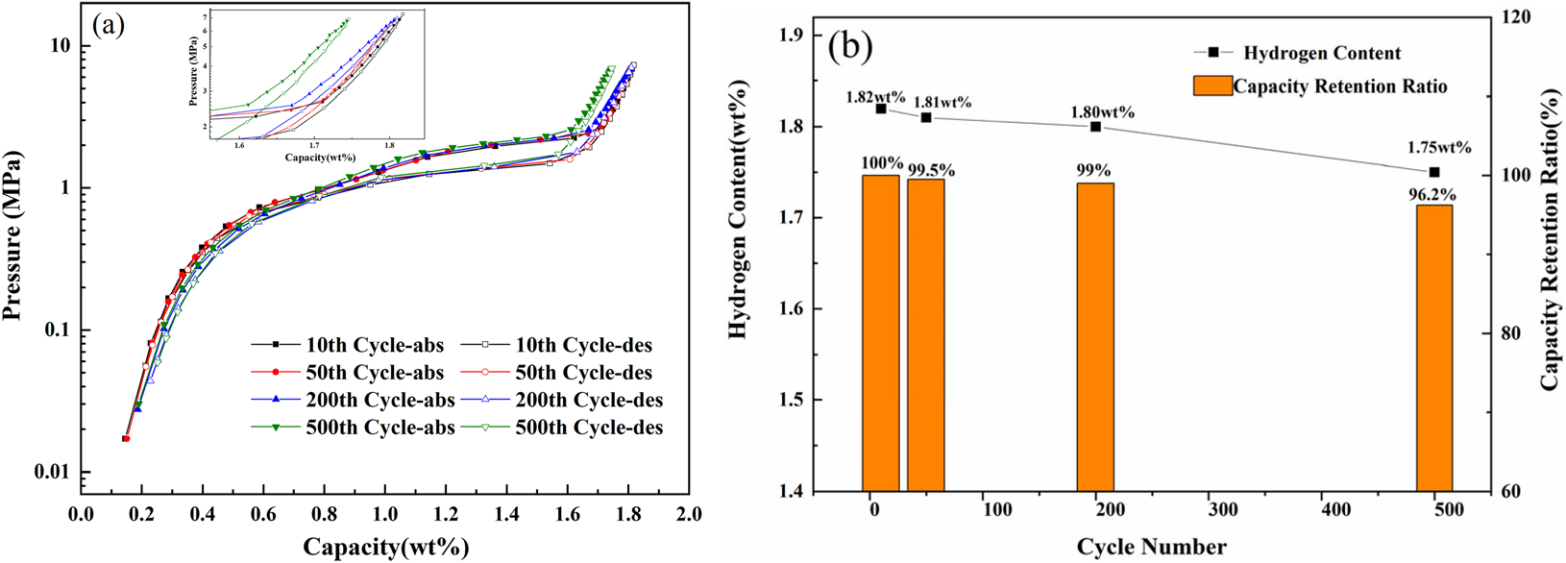
(a) PCT curves of the Ti_0.84_Zr_0.16_Mn_0.9_Cr_0.7_Fe_0.1_ alloy after 10, 50, 200, and 500 cycles. (b) Changes in hydrogen storage capacity during various cycles.

Fig. 11 shows the PCT curves and hydrogen capacity maintenance plots of the Ti_0.84_Zr_0.16_Mn_0.9_Cr_0.7_Fe_0.1_ alloy at 313 K for different number of cycles. The hydrogen storage capacity gradually decreased from 1.818 wt% at the 10th cycle to 1.809 wt% at the 50th cycle, 1.807 wt% at the 200th cycle, and finally to 1.745 wt% at the 500th cycle. After 500 cycles of hydrogen absorption and desorption, the alloy exhibited good cycling stability with 96.2% capacity maintenance. As shown in Table 5, the cycling stability of several Ti–Mn-based alloys is compared, among which the Ti_0.84_Zr_0.16_Mn_0.9_Cr_0.7_Fe_0.1_ alloy exhibits superior capacity retention. As can be seen from Fig. 9, the crystal structure of the alloy remained unchanged, maintaining a single C14 crystal structure without the appearance of a second phase. According to previous studies by Gamo,^48^ Ti–Mn based alloys did not cause disproportionation during cycle tests and were degraded by reaction with impurities in hydrogen gas. Tatsuo *et al.*^49^ investigated the effect of gas purity on the cycling performance of Ti_0.93_Zr_0.07_Mn_1.15_Cr_0.35_ and found that the capacity maintenance of the alloy after 2000 cycles was 81.8% under hydrogen purity of 99.99%, and 97.2% under hydrogen purity 99.99999%. Nitrogen, as an impurity in hydrogen, reacts with the alloy and accumulates, leading to a reduction in the hydrogen storage sites and a decrease in the alloy's capacity. Shahrouz *et al.*^50^ proposed that the position of Mn atoms in Ti–Mn-based alloys undergoes structural changes as the composition of the C14 Laves phase changes during hydrogen cycling. This could result in retained hydrogen inside the Laves phase, with its scale gradually increases. In this study, the capacity maintenance rate after 500 cycles at 99.999% hydrogen purity was 96.2%, and it could be concluded that the capacity attenuation of the alloy might be due to hydrogen purity.

**Table 5:** Cycling properties of several Ti–Mn based alloys

Alloy	Cycle number	Capacity retention ratio	References
Ti_0.84_Zr_0.16_Mn_0.9_Cr_0.7_Fe_0.1_	500	96.2%	This work
Ti_0.8_Zr_0.2_Mn_0.9_Cr_0.6_V_0.3_Fe_0.2_	10	95%	51
TiZrFeMnCrV	50	97.8%	52
Ti_0.9_Zr_0.1_V_0.45_Mn_1.3_Cr_0.2_	200	95.2%	53

## 4. Conclusions

The non-stoichiometric Ti–Mn-based Ti_0.84_Zr_0.16_Mn_0.9+*x*_Cr_0.7_Fe_0.1_ (*x* = 0, 0.1, 0.2) and Ti_0.84_Zr_0.16_Mn_0.9_Cr_0.7+*y*_Fe_0.1_ (*y* = 0, 0.1, 0.2, 0.3) alloys were prepared and investigated, including their microstructure, hydrogen storage properties, and cycling stability. We concluded that:

(1) The non-stoichiometric alloys, with various Mn and Cr contents, all crystallized in the single C14-type Laves phase structure. As the Mn and Cr content or Mn/Cr ratio increased, the cell volumes of the C14 phase decreased.

(2) With the increase in Mn or Cr content, the hydrogen storage capacities decreased, and the equilibrium hydrogen absorption/desorption pressures increased. Meanwhile, the hysteresis coefficient increased, and the slope of the plateau phase decreased. As the Mn/Cr ratio decreased, the hysteresis of the alloys improved significantly, while the plateau pressures and hydrogen storage capacities decreased slightly.

(3) The increase in Mn and Cr content worsened their hydrogen absorption kinetics, whereas the alloys with smaller stoichiometric ratios exhibited a faster hydrogen absorption rate. In all cases, the kinetic properties were governed by the rate-controlling step of hydrogen diffusion forming metal hydride.

(4) The Ti_0.84_Zr_0.16_Mn_0.9_Cr_0.7_Fe_0.1_ alloy showed excellent cycling performance, with 96.2% hydrogen storage capacity retention after 500 hydrogen absorption and desorption cycles.

## Data availability

All data included in this study are available upon request by contacting the corresponding author.

## Conflicts of interest

There are no conflicts to declare.
